# A New Calcium Oral Controlled-Release System Based on Zeolite for Prevention of Osteoporosis

**DOI:** 10.3390/nu11102467

**Published:** 2019-10-15

**Authors:** Angela Fabiano, Anna Maria Piras, Vincenzo Calderone, Lara Testai, Lorenzo Flori, Dario Puppi, Federica Chiellini, Ylenia Zambito

**Affiliations:** 1 Department of Pharmacy, University of Pisa, via Bonanno 33, 56126 Pisa, Italy; angela.fabiano@unipi.it (A.F.); anna.piras@unipi.it (A.M.P.); vincenzo.calderone@unipi.it (V.C.); lara.testai@unipi.it (L.T.); lorenzoflori17@gmail.com (L.F.); 2 Interdepartmental Research Center Nutraceuticals and Food for Health, University of Pisa, via Borghetto 80, 56124 Pisa, Italy; 3 Department of Chemistry and Industrial Chemistry, University of Pisa, Udr INSTM Pisa, Via Moruzzi 13, 56124 Pisa, Italy; dario.puppi@unipi.it (D.P.); federica.chiellini@unipi.it (F.C.)

**Keywords:** calcium zeolite, Precirol^®^ granules, Calcium controlled release, osteopenic rats, femur mechanical characterization, femur morphological characterization

## Abstract

Osteoporosis, a systemic skeleton disease, can be prevented by increasing calcium levels in serum via administration of calcium salts. However, traditional calcium-based formulations have not appeared to be effective, hence the purpose of the present work has been to prepare and test in vitro/vivo a formulation able to gradually release calcium during transit over the GI tract, thus increasing bioavailability and reducing daily dose, and hence, side effects. Calcium controlled-release granules based on zeolite and Precirol^®^ were prepared. In the best case, represented by granules sized 1.2 mm, containing 20% Precirol^®^, 19% zeolite, 60% calcium (granule), the release lasted ≈6 h. The release is controlled by diffusion of calcium ions through the aqueous channels forming within granules, once these come into contact with physiological fluids. Such a diffusion is hindered by the interaction of calcium ions with the negatively charged surface of the zeolite. Ovariectomy was used to make rats osteopenic. For in vivo studies, rats were divided into the following groups. Sham: not treated; ova: ovariectomized (ova); CaCl_2_ 1.0 g: ova, treated with 1.0 g/die Ca^2+^; CaCl_2_ 0.5 g: ova, treated with 0.5 g/die Ca^2+^; granule 1.0 g, or granule 0.5 g: ova, treated with granules equivalent to 1.0 g/die or 0.5 g/die Ca^2+^ in humans. Ca^2+^ amounts in femur bone and bone marrow, femur mechanical characteristics, and femur medullary canalicule diameter were measured and the same efficacy rank order was obtained: ova < CaCl_2_ 0.5 g < CaCl_2_ 1.0 g < granule 0.5 g ≈ granule 1.0 g ≈ sham. The results show promise of an effective prevention of osteoporosis, based on a controlled-rate administration of a calcium dose half that administered by the current therapy, with reduced side effects.

## 1. Introduction

Osteoporosis is a systemic skeleton disease, characterized by a reduction of bone mass and a deterioration of bone tissue microarchitecture, with consequent increase of frailty and liability to fracturing, mostly of hip bone, back bone, or wrist bone. [1,2,3]. According to the World Health Organization (WHO), almost 40% of women aged over 50 in western populations are suffering from various degrees of osteoporosis. To prevent osteoporosis, increasing serum calcium levels has been attempted by administering adequate calcium amounts [4,5,6].

The most common calcium supplements currently in use are carbonate, citrate, chloride, and gluconate calcium salts. The known calcium formulations do not allow a gradual release of calcium ion, in fact, release is only determined by the salt solubility in gastrointestinal fluids. However, a therapy based on administration of calcium salts has shown various contraindications caused by hypercalcemia, which may cause nausea, vomiting, constipation, abdominal pain, thirst, polyuria, polydipsia, hypertension and vasomotor disorders, hypercalciuria, calcium lithiasis, and severe renal failure. Calcium supplements with or without vitamin D are useful, even essential, for those whose diet cannot include dairy products. Calcium is actively absorbed in duodenum and proximal jejunum via carriers, the synthesis of which is stimulated by vitamin D. Furthermore, it is absorbed passively via the paracellular route in the whole intestine, including, though minimally, the colon [7]. Calcium transport via the paracellular route is not influenced by vitamin D, rather, it only depends on calcium solubility, chimo transit time over the intestine, and calcium paracellular permeability [8].

Such calcium salts as phosphates and carboxylates are poorly soluble in water, where they form nonamphotheric hydroxides. The calcium ions that are formed in the stomach by acid dissolution can precipitate as hydroxides when the partially digested chimo is transferred to the intestine, where the environment is increasingly alkaline. Calcium precipitation may be prevented by the formation of soluble complexes. Calcium precipitation from supersaturated solutions, resulting from neutralization of chimo pH in the intestine, can also be inhibited by kinetic means, enhancing calcium bioavailability. Precipitation inhibitors or complexing agents must be found in the intestine. The absence of such agents in this phase of digestion can result in calcium precipitation and bioavailability reduction, with detrimental consequences on health [9,10,11].

The present work is mainly aimed at preparing and testing a formulation able to gradually release calcium during gastrointestinal transit, thus improving calcium bioavailability, thus reducing calcium daily supplementation and side effects. Indeed, a controlled-release formulation can release calcium in the intestine at such a rate as to replace the amount absorbed without increasing calcium concentration up to values higher than the maximum free calcium ion concentration allowed in the intestine [12]. Moreover, a calcium supplementation concurrent with a calcium rich diet may cause hypercalcemia, which is responsible for many side effects, the most serious of which affect the heart. A controlled-release formulation could prevent such effects. 

A formulation based on zeolites has been prepared and tested in the present work. Zeolites have been studied for different pharmaceutical applications, e.g., as vehicles of low molecular mass active principles, such as nonsteroidal anti-inflammatory drugs [13,14], or their ion exchange characteristics and antimicrobial properties [15,16]. Calcium controlled-release granules based on zeolite and Precirol^®^ were prepared and tested on osteopenic rats. They are also the object of a patent application [17].

## 2. Materials and Methods

### 2.1. Preparation of Calcium-Containing Zeolite Granules

The procedure for loading zeolite powder (clinoptilolite, average diameter <50 µm; Glencor S.r.l.) (Z) with Ca^2+^ ions involved pouring zeolite powder in water at a 5% weight/volume concentration, to give an aqueous suspension, to which an equal volume of an almost saturated CaCl_2_ aqueous solution was subsequently added. The resulting suspension was stirred for a few hours. Subsequently, the suspension was centrifuged, and the sediment, after drying at 40 °C under vacuum, was used to prepare the nutraceutical product. The supernatant was analyzed for Ca^2+^, in order to determine the percentage of Ca^2+^ encapsulated in the zeolite. To prepare the final granules of the compound, a mix of dried sediment and a waxy excipient (Precirol^®^ ATO 5, Gattefossé) (P) was heated to 75 °C and extruded to form granules of 0.9 or 1.2 mm size. The granule composition and size are listed in Table 1. 

### 2.2. Study of Calcium Release from Granules

The elution medium was comprised of simulated gastrointestinal fluids, such as the following: simulated gastric fluid (SGF), comprised of HCl 0.04 M, pH 1.2, made isotonic with NaCl (40 g HCl 1 N plus 1 g NaCl per 500 mL). An isotonic NaCl solution (500 mL), the pH of which was monitored during the release runs and controlled to 6.8 or 7.4 by adding drops of concentrated NaOH, was used to simulate jejunal fluid (SJF) or simulate large intestine environment (SLIE), respectively. To measure Ca^2+^ release from granulations, a test was carried out in which at time t = 0, a stirrer (60 rpm) was immersed in the receiving phase (100 mL containing 100 mg of the granulation under test) [18]. The fluids used to simulate the intestinal environment contain no ions that could form any insoluble calcium salts; therefore, the experimental conditions used are believed to allow an as high release rate as possible. Sample analysis was carried out fluorometrically, using Fura-2, which is considered one of the most effective and selective Ca^2+^ indicators [19].

### 2.3. Characterization of Granules by ATR-FTIR Spectroscopy

The components of the formulation under study, either alone or in the form of mixtures or granulates, were characterized by ATR-FTIR (Attenuated Total Reflectance-Fourier Transform InfraRed) spectroscopy (Cary 660 series FTIR, Agilent Technologies, Milano, Italy). Before analysis, they were vacuum dried overnight at 37 °C. The spectra were acquired in the 650–4000 cm^−1^ range, with a resolution range of 4 cm^−1^ and 64 scans. 

### 2.4. Experiments In Vivo

In vivo experiments were carried out according to European (EEC Directive 2010/63) and Italian (D.L. 4 March 2014 n.26) legislation (protocol number: 134/2019-PR, 14/02/2019); moreover, ARRIVE guidelines have been put into practice [20]. Thirty-six animals were housed in cages with free access to standard food pellets and water under standard conditions (12-hour light/dark cycle, 22 °C). Three-month-old female Wistar rats (200–250 g) were used for these experiments. They were subjected to ovariectomy. Ovariectomized female rats are considered a reliable model that reproduces the clinical conditions typical of menopause, including osteopenia and osteoporosis [21].

The animals were anesthetized with thiopental sodium (60 mg/Kg i.p.), placed on prewarmed operating tables, and subjected to surgery. Following surgery, the animals were individually placed in cages, where they were subjected to the appropriate analgesic and antibiotic treatment, according to the veterinary indications. After 7 days, the animals were randomly assigned to the following experimental groups, each composed of 6 animals.

- Sham Group: nonovariectomized rats not treated with calcium formulations;
- Ova Group: ovariectomized rats not treated with calcium formulations;
- CaCl_2_ 1.0 g Group: ovariectomized rats treated with 200 mg/Kg/die CaCl_2_, equivalent to a 1.0 g/die Ca^2+^ dose in humans;
- CaCl_2_ 0.5 g Group: ovariectomized rats treated with 100 mg/Kg/die CaCl_2_, equivalent to a 0.5 g/die Ca^2+^ dose in humans;
- Granule 1.0 g Group: ovariectomized rats treated with 130 mg/Kg/die granules, equivalent to a 1.0 g/die Ca^2+^ dose in humans;
- Granule 0.5 g Group: ovariectomized rats treated with 65 mg/Kg/die granules, equivalent to a 0.5 g/die Ca^2+^ dose in humans.

Calcium formulations mixed with food, purchased from Rettenmaier, Mantova, Italy, were administered once a day per os, by gavage, and the treatment was continued for 8 weeks. The animals were monitored daily for food and water intake, and body weight was measured, in order to gain useful information on their health. At the end of the experimental protocol, the animals were sacrificed, and the beneficial effects of the treatment on the bone structure of femurs were evaluated, according to a procedure that will be described in the next section. Blood samples were collected by means of an intracardiac puncture for dosage of osteocalcin (OC).

### 2.5. Determination of Calcium Concentration in Bone and Bone Marrow

The bone marrow was aspirated from each femur by a syringe, while the bone, after removing all adherent tissue components, was washed with ethanol and vacuum dried 12 h at 37 °C. An accurately weighed dry femur amount (≈30 g) was crushed, dissolved in 3 mL PBS made to pH ≈ 3 with HCl 1 N, and stirred for 1 h at room temperature [22]. Subsequently, the sample was centrifuged (10,000 rpm, 5 min) and the supernatant analyzed for Ca^2+^ content. After aspiration, the bone marrow was dispersed in 1 mL PBS made to pH 3 with HCl 1 N, and homogenized by Turrax^®^ (8000 rpm, 10 min) [23,24]. The homogenized product was then centrifuged (10,000, 5 min), and the supernatant was analyzed for Ca^2+^ content. The results are expressed as Ca^2+^ mg/g bone or bone marrow.

### 2.6. Rat Femur Mechanical Characterization

Bone femur specimens were subjected to three-point bending in the anteroposterior direction using an Instron 5564 uniaxial testing system (Instron, Norwood, MA, USA) with a 2 kN load cell. Bearing- and loading-bars had a rounded tip with a diameter of 2.5 mm, and the distance between the bars was adapted according to each specimen length. Five replicates of each kind of bone sample were tested by transmitting the force at the middle position between the distal and proximal site with a constant test velocity of 5 mm/min until failure. A load–displacement diagram was obtained from a software recording data (Merlin). The stiffness was defined as the slope of the linear region in the force/displacement graph, and the breaking load was defined as the maximum load before breaking.

### 2.7. Rat Femur Morphological Characterization

The central parts of the femoral diaphyses obtained from the mechanical strength tests, were cut into 3 mm segments each and depleted of bone marrow, using a plastic syringe equipped with a 22G needle, followed by repeated washing with deionized water and ethanol, and overnight vacuum drying at 37 °C. Afterwards, the specimens were further cut longitudinally and the external surface or the medullary cavities were morphologically characterized by means of field emission—scanning electron microscope (FE-SEM; Quanta 450 ESEM FEG, FEI, Hillsboro, OR, USA). Canalicular diameters were measured as the smallest size of the opening and were evaluated from 6 random fields (Image J 1.50i; Wayne Rasband National Institute of Health, Bethesda, MD, USA). The average size was assessed by micrographs at 8000 magnification. The image was divided into 150 µm^2^ regions by applying a grid, and the mean diameter of the canalicular openings was evaluated by over 50 measurements per specimen taken from randomly selected fields.

### 2.8. Statistical Analysis

The GraphPad Prism Software vs. 7.0 (GraphPad Sftware Inc. La Jolla, San Diego, CA, USA) was used for the statistical analysis of data. All results are presented as means ± standard deviation of at least six independent experiments. The difference between means was evaluated by the Student’s *t*-test. Differences were considered significant, i.e., the null hypothesis was rejected, for *p*-values lower than 0.05.

## 3. Results and Discussion

Calcium releasing granules were prepared under dry conditions by extrusion through either 0.9 mm or 1.2 mm sized sieves. Zeolite was first loaded with calcium chloride, followed by mixing and extrusion with Precirol^®^. Two main zeolite/precirol ratios were investigated, namely 80:20 or 50:50 by weight (Table 1). In order to assess the effect of the formulation components, alternative formulations were investigated such as: formulations coded C:P, only containing calcium as a second component (without zeolite); formulations coded (C+T):P, where the zeolite was replaced by talc, an inert component having a chemical composition similar to that of zeolite.

### 3.1. Calcium Release from Granules

**Figure 1:**
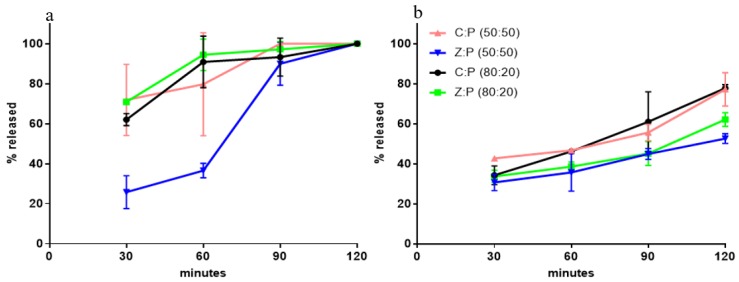
Ca^2+^ fraction released from granules immersed in simulated gastric fluid (SGF): (**a**) size, 0.9 mm; (**b**) size, 1.2 mm.

Calcium release in SGF was evaluated, and the comparison between the formulations tested allowed for the selection of the best granule composition to be submitted to the in vivo evaluations. Figure 1a shows the profiles of the Ca^2+^ fraction released over 120 min, from 0.9 mm granules to SGF. It is seen from the data, that in all cases the release is virtually complete in 90 min. Figure 1b shows the Ca^2+^ fraction released from granules sized 1.2 mm to SGF. It is seen that the release is slowed down and influenced by the zeolite. Indeed, in the presence of zeolite, the release over the first 2 h is unaffected by the Z/P ratio. For this reason, the formulation containing the highest calcium dose (Z:P (80:20)) was further investigated, also considering that the calcium dose in commercial products is as high as 1000 mg/die. The difference between data shown in Figure 1a,b proves the dependence of the release rate on granule size. Such a difference also suggests that the release rate is controlled by calcium ions diffusion in aqueous channels within granules.

**Figure 2:**
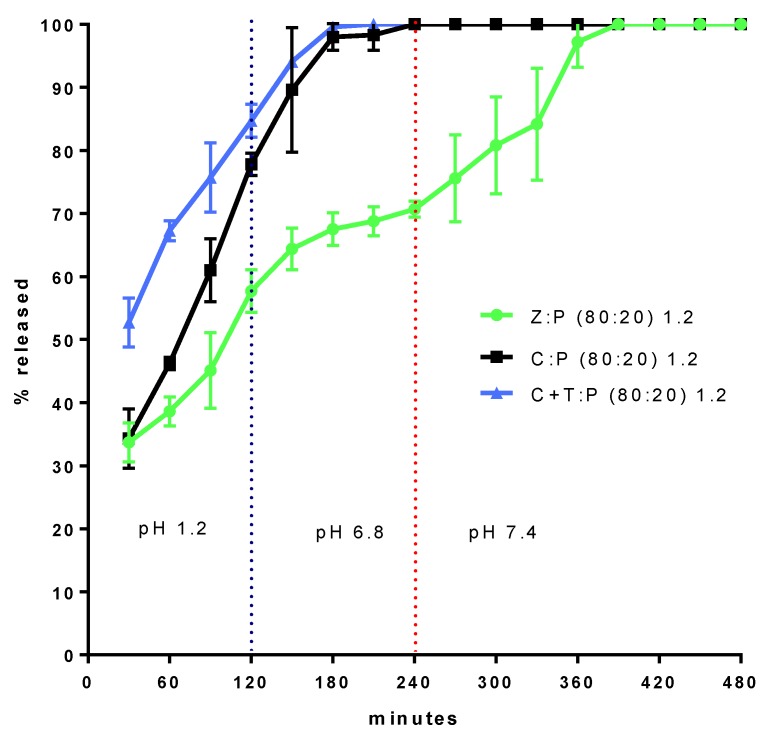
Ca^2+^ fraction released from granules 1.2 mm size immersed, in sequence, in SGF, simulate jejunal fluid (SJF), and then in simulate large intestine environment (SLIE).

Figure 2 shows the Ca^2+^ fraction released from granules of 1.2 mm size immersed, in sequence, in SGF for 120 min, SJF for 120 min, and then in SLIE. The Z:P (80:20) 1.2 (granule) formulation was subjected to simulated intestinal digestion (pH 6.8 and 7.4) together with two formulations, taken as references, both with the same 20% P proportion.

A formulation, coded C:P (80:20) 1.2, only contains calcium as a second component, another contains talc (19%) and calcium (61%) and is coded (C+T):P (80:20) 1.2. Figure 2 shows the extent to which the zeolite is able to delay calcium release, with respect to the two reference formulations. Indeed, the zeolite-containing formulation gives rise to a continuous and gradual calcium release for about 6 h, while in the absence of zeolite, calcium release is complete in 3 h. From the release profile obtained with the formulation Z:P (80:20) 1.2, it can be hypothesized that the release is controlled by the diffusion of calcium ions through the aqueous channels forming within granules, once these come into contact with physiological fluids. Such a diffusion is hindered by the interaction of the calcium ions with the negatively charged surface of the zeolite. Indeed, the granules comprised of the sole calcium and P (C:P (80:20) 1.2) are unable to control the release. The data also show that the zeolite molecular nanoporous structure, thanks to the high specific surface available for the interaction with calcium ions, is indispensable for release control. Indeed, the talc containing granules ((C+T):P (80:20) 1.2) appear unable to control the release. However, were the release controlled by the sole diffusion of calcium ions in the zeolite aqueous channels, the release rate would decrease over time, which is not observed in the profile relative to Z:P (80:20) 1.2. In fact, this formulation shows an increase in release rate after 4 h from the beginning of the experiment. This is explained by a progressive disintegration of granules as they become depleted of calcium.

### 3.2. Granule Characterization by ATR-FTIR Spectroscopy

**Figure 3:**
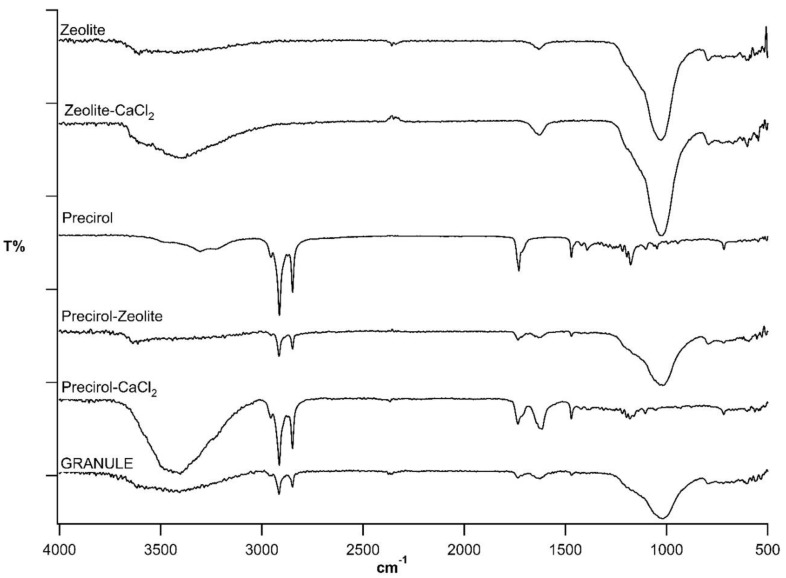
Attenuated Total Reflectance-Fourier Transform InfraRed (ATR-FTIR) spectra of Z:P (80:20) 1.2 (GRANULE) and formulation components.

Spectra for the components of the formulation under study are compared with that for the granules Z:P (80:20) 1.2 in Figure 3, and the relevant data are listed in Table 2. It should be considered that zeolite, an aluminum silicate, shows both Si–OSi (Al) stretching signals (1027 and 795 cm^−1^) and H–O–H hydration bands (3600–3400 cm^−1^). Zeolite–CaCl_2_ association brings about no significant change of zeolite spectrum.

The characteristic bands of Precirol^®^ are due to its aliphatic chains (2956–2848 cm^−1^, asymmetrical and symmetrical stretching; 1471 and 1392, asymmetrical and symmetrical bending) and its ester carbonyls (1730 cm^−1^). The association of Precirol^®^ with CaCl_2_ does not significantly modify the wax spectrum, whereas the spectrum of the Precirol^®^–zeolite mix shows the signals of both components. The characteristic signals of the single components, i.e., zeolite, CaCl_2_ (silicate stretching, hydration associated water), and Precirol^®^ (aliphatic stretching and bending, ester carbonyl stretching) can be recognized in the spectrum of the finished granules.

### 3.3. Experiments In Vivo

In the course of in vivo experiments, carried out under the constant supervision of a veterinarian, the animals gave no signs of suffering, toxicity, or behavioral or vegetative alterations.

#### 3.3.1. Determination of Calcium Concentration in Rat Bone and Bone Marrow

**Figure 4:**
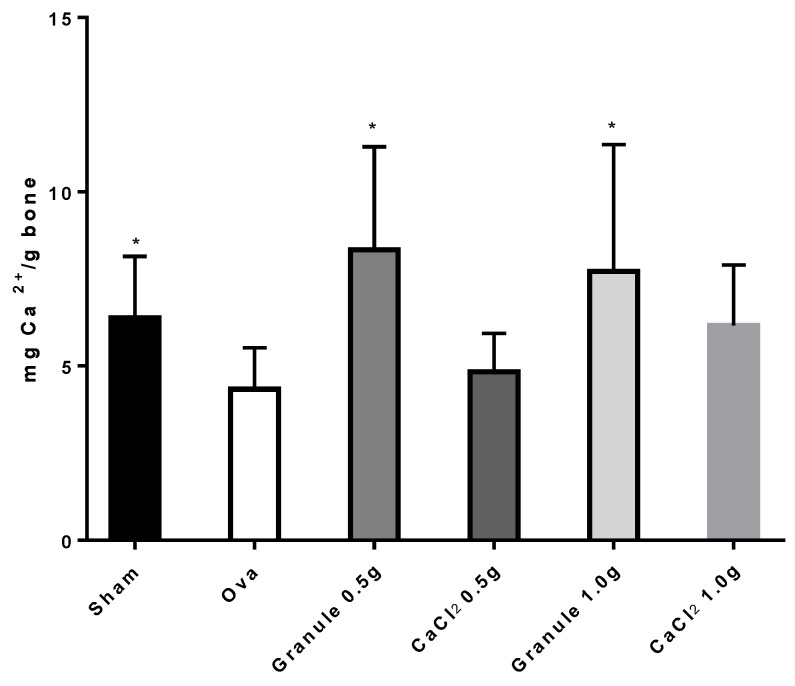
Ca^2+^ quantity (mg) per g of bone in rat femur. *p < 0.05 versus Ova Group.

The experimental protocol followed in the present work is known to cause osteopenia, not osteoporosis in rats [21]. Indeed, to develop osteoporosis after ovariectomy, rats must be subjected to a diet poor in calcium for several weeks [25]. Figure 4 shows data for the Ca^2+^ amount per gram of femur bone following treatment of the different rat groups. According to the data, the administration of the sole CaCl_2_ improved the calcium content in bone, although without any significant differences from the ova group. Diversely, rat treatment with granules could make the Ca^2+^ amount in bone to values significantly different from the ova group, though not significantly different from the sham group. Although the Ca^2+^ amount in bone increases with increasing CaCl_2_ dose administered, no differences are observed between rats treated with different Ca^2+^ doses in granules. Hence, a calcium dose in granules higher than 0.5 g produces no further Ca^2+^ increase in bone.

**Figure 5:**
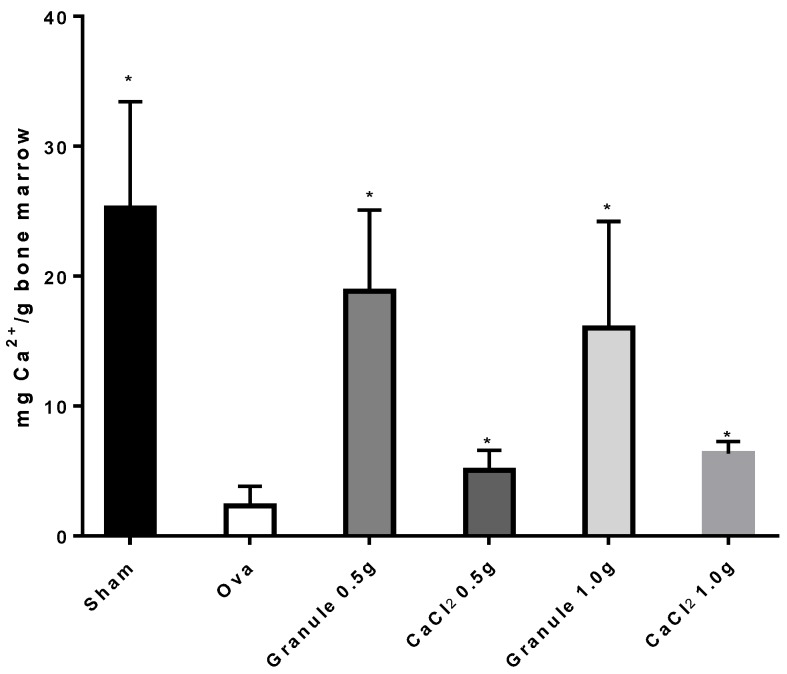
Ca^2+^ amount (mg) per g of bone marrow in rat femur. *p < 0.05 versus Ova Group.

Data for the Ca^2+^ mg/g bone marrow taken from femur following treatment of the different rat groups are shown in Figure 5. The treatment with two different doses of calcium salts significantly increased the Ca^2+^ amount in femur bone marrow, though it did not reach the value found in the sham group. As Figure 5 shows, the amount of calcium found in the bone marrow of rats treated with granules was about three-fold higher than that found in rats treated with CaCl_2_ salts, and about eight-fold higher than that found in the ova group. Conversely, no significant difference in calcium content in bone marrow was observed between the sham group and the two granule groups, which clearly indicates that the granule formulations are able to bring the calcium levels in bone marrow back to values found in healthy rats. Concerning the treatments with CaCl_2_ in salt form, calcium levels in bone marrow increased with respect to the ova group but remained far lower than those found in either sham or granule-treated rats.

These data altogether point to a faster accumulation of calcium into the osteogenic cellular types within the bone marrow. Indeed, bone marrow is generally considered a rich source of fat, proteins, and minerals, including calcium [26]. High levels of calcium can be correlated with the cellular compartment of bone marrow, in particular to the osteogenic cell types containing bone matrix vesicles (BMV). These vesicles have recently been recognized as key players in bone mineralization processes and calcium homeostasis. In particular, BMV are specialized structures that allow for the confinement and accumulation of calcium and phosphate ions within chondrocytes, osteoblast, and preosteoblast cell types. BMV lumen is rich in proteins capable of chelating calcium ions, allowing for a massive increase of intracellular calcium concentration with respect to the extracellular matrix and blood concentrations [27,28,29]. Furthermore, the importance of BMV in bone marrow has been underlined by the possibility of exploiting BMP-bound alkaline phosphatase (ALP) as a marker for bone marrow (BM) aspirates in diagnostics and for research purposes [30].

#### 3.3.2. Rat femur Mechanical Characterization

Data for the mechanical characteristics of femurs of the different rat groups are listed in Table 3. Although no significant difference is seen between groups for stiffness or breaking load, data follow the same rank order (ova < CaCl_2_ 0.5 g < CaCl_2_ 1.0 g < granule 0.5 g ≈ granule 1.0 g ≈ sham) as that already found for calcium content in either femur or bone marrow. The lack of difference significance is probably due to our experimental protocol implying the use of osteopenic, not osteoporotic rats [21]. In fact, not even significant differences between sham and ova groups could be evidenced. In any case, data validate the thesis of a greater ability of the controlled-release granules than the traditional formulation to prevent osteoporosis.

#### 3.3.3. Rat Femur Morphological Characterization

**Figure 6:**
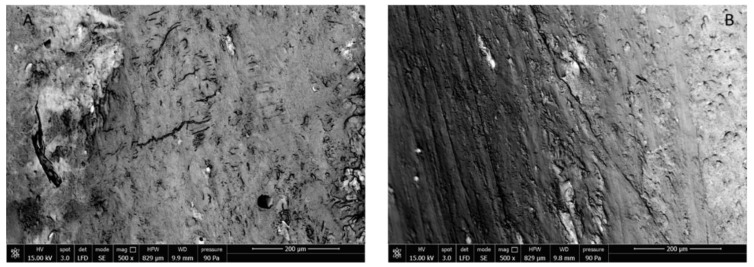
SEM images of external surface of sham (**A**) and ova (**B**) rat femur.

SEM images of sham rat and ova rat femurs are shown in Figure 6. Significant differences between surfaces due to either cavitation or flaws cannot be discerned in the images, which confirms that the protocol had not caused osteoporosis in ova rats.

For this reason, SEM images of the medullary part of femur were also taken. Indeed, as it is known, ovariectomized rats are characterized by a higher bone lacunar-canalicular porosity than healthy rats [31]. 

**Figure 7:**
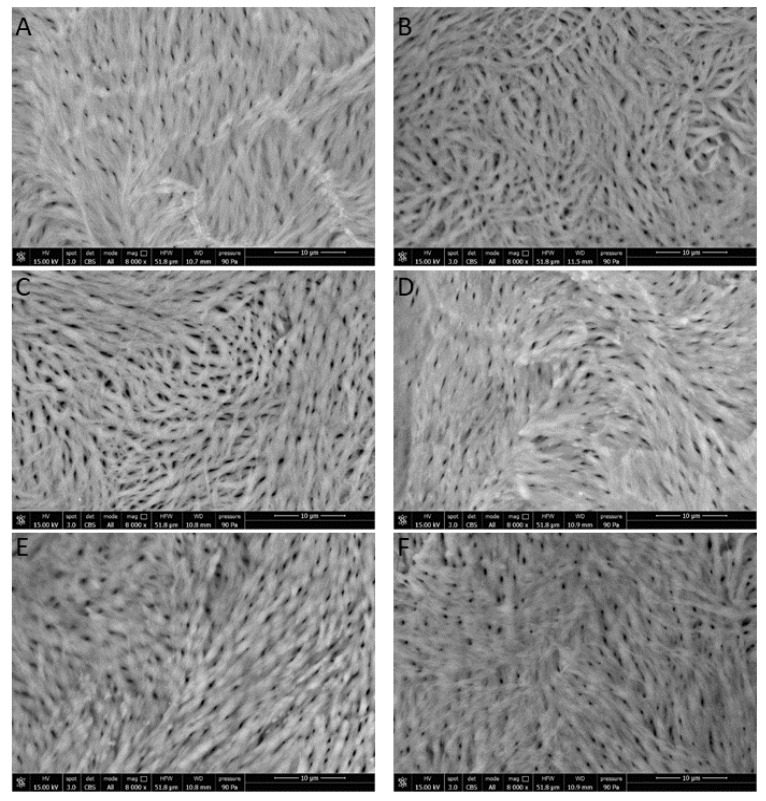
SEM images of inner rat femur: (**A**) sham; (**B**) ova; (**C**) CaCl_2_ 0.5 g; (**D**) CaCl_2_ 1.0 g; (**E**) Granule 0.5 g; (**F**) Granule 1.0 g.

**Figure 8:**
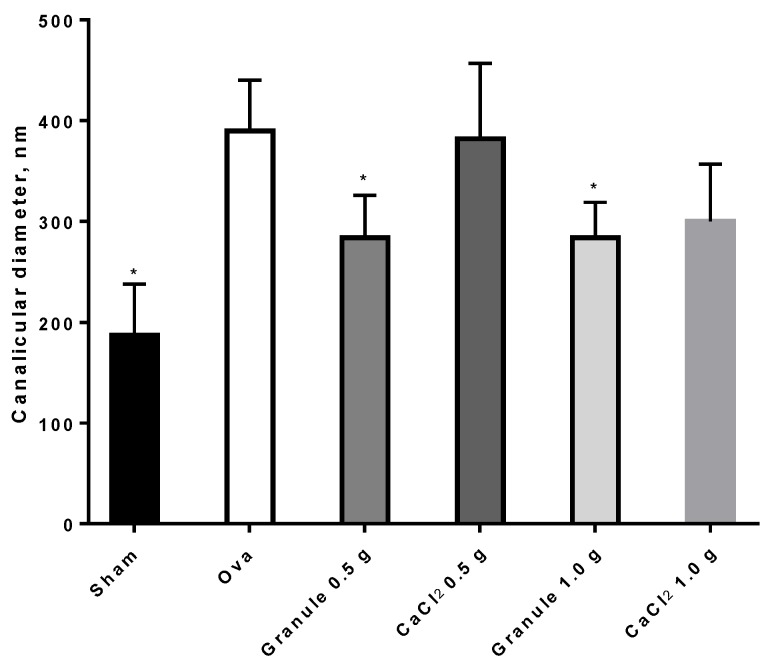
Canalicular diameters ± SD of rat femurs. *p < 0.05 versus Ova.

Images appearing in Figure 7 show a difference in femur canalicules diameter between sham (Figure 7A, smaller diameter) and ova (Figure 7B, larger diameter). The determination of the mean canalicule size from the images of Figure 7 concerning rat femurs from the different treatment groups was attempted. The results, shown in Figure 8, indicate that only in Sham rats and in those treated with the controlled-release granules was the mean canalicule size significantly smaller than that measured in ova rats. Then the traditional therapy, based on calcium salts, has appeared less effective, in preventing osteoporosis, than the present one based on the controlled-release system.

#### 3.3.4. Plasmatic Marker of Osteopenia/Osteoporosis

Osteocalcin (OC) is a main noncollagenous protein involved in bone matrix organization and deposition. Indeed, OC is produced during bone formation, in the late mineralization process, and can directly and indirectly control mass, mineral size, and orientation. Knock-out mice for OC present a loss of bone toughness and a colocalization of this protein with microdamage accumulation, indicative of its important role in determining bone size, shape, and strength [32].

**Figure 9:**
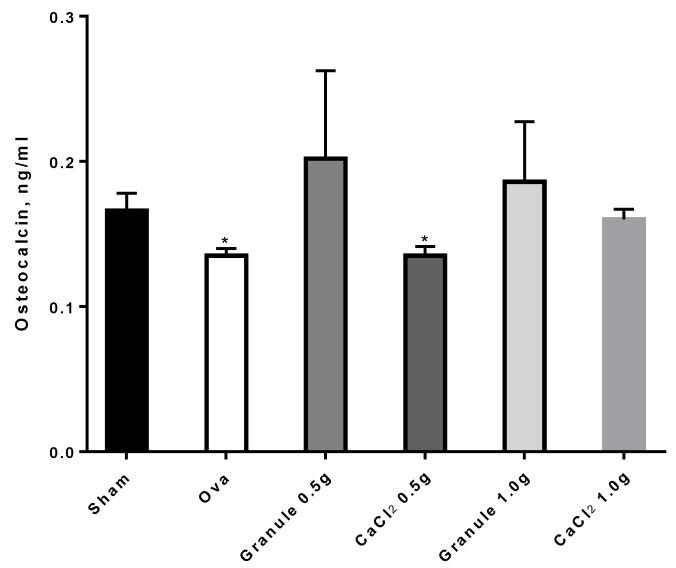
Osteocalcin levels in the different experimental groups. *p < 0.05 versus sham.

In particular, from the data in Figure 9 it can be seen that ova rats showed OC levels significantly lower than sham rats, in agreement with relevant literature reports. On the other hand, the higher dose (1 g/Kg) of the traditional calcium formulation increased OC significantly, i.e., up to levels comparable to those for sham rats. It is worth noting that granule rats, both at 0.5 and 1.0 g/Kg, despite the data variability, showed increased OC levels, up to higher values than those for the sham group, thus confirming the encouraging results reported above.

## 4. Conclusions

Zeolites allowed us to obtain a Ca^2+^ controlled release formulation. Negative charges are found on the surface of such silicates that bind the calcium positive ions via ionic exchange with the zeolite negative ions. In vivo experiments carried out on osteopenic rats demonstrated the ability of the patented controlled-release formulation to increase the calcium content in the bone and bone marrow and decrease the canalicular diameter of the rat femur. The dose equivalent to 0.5 g/Kg/die in humans has appeared the maximum effective dose in granules, beyond which no increase in effectiveness has been seen. The results show promise of an effective prevention of osteoporosis based on a controlled-rate administration of a calcium dose half that administered by the current therapy, with reduced side effects. As we cannot rule out that a dose lower than 0.5 g/Kg/die may be effective, our aim for the near future will be to carry out a study on the possibility of further reducing the dose. Furthermore, it will be interesting to study the effectiveness of the present system on osteoporotic rats, in order to understand if the calcium controlled-release granules are able to heal osteoporosis in addition to preventing it.

## 5. Patents

Zambito Ylenia, Fabiano Angela, Piras Anna Maria. Oral controlled-release calcium compound and method for preparing it. 2018; European Patent No EP3482745.

## Figures and Tables

**Table 1:** Characteristics of granules.

Code	Precirol %	Ca^2+^ %	Zeolite %	Talc %	Granule Size (mm)
Z:P (80:20) 0.9	20	61	19		0.9
Z:P (50:50) 0.9	50	37.8	12.2		0.9
C:P (80:20) 0.9	20	80			0.9
C:P (50:50) 0.9	50	50			0.9
Z:P (80:20) 1.2 ^a^	20	61	19		1.2
Z:P (50:50) 1.2	50	37.8	12.2		1.2
C:P (80:20) 1.2	20	80			1.2
C:P (50:50) 1.2	50	50			1.2
(C+T):P (80:20) 1.2	20	61		19	1.2

^a^ The formulation Z:P (80:20) 1.2 was used for the in vivo studies and named “Granule”.

**Table 2:** Bands H–O–H of hydration associated water. Signals for the: stretching (symmetrical, v_s_ and asymmetrical, v_as_) of Si–O and C–H bonds; bending (symmetrical, δ_s_ and asymmetrical, δ_as_) of C–H bond; stretching of ester carbonyl.

Components	Hydration H_2_O	ν_as_ C–H, ν_s_ C–H			ν_s_ C=O	Hydration H_2_O	δ_as_ C–H	δ_s_ C–H	Silicate ν_as_ e ν_s_ Si–O	
Zeolite	3600					1632			1027	795
Zeolite CaCl_2_	3400					1631			1027	795
Precirol^®^	3304	2955	2913	2848	1730		1471	1392		
Precirol^®^ Zeolite	3600	2955	2915	2849	1734	1634	1471	1392	1027	795
Precirol^®^ CaCl_2_	3403	2956	2914	2849	1734	1623	1471	1392		
GRANULE	3400	2960	2916	2848	1735	1626	1470	1396	1020	798

**Table 3:** Stiffness and breaking load data for rat femurs. Means ± SD.

Rat Groups	Stiffness (N/mm)	Load Max (N)
Sham	159 ± 17	107 ± 6
Ova	113 ± 24	93 ± 18
Granule 0.5 g	136 ± 38	106 ± 9
CaCl_2_ 0.5 g	118 ± 51	101 ± 13
Granule 1.0 g	136 ± 19	101 ± 7
CaCl_2_ 1.0 g	128 ± 11	93 ± 11
